# Supplementary material for: Three-year of hepatocellular carcinoma surveillance in patients with cirrhosis diagnosed between 2009 and 2013: a cohort study based on the French National Health Data System (SNDS) claims data
Source: Front Oncol. 2025 Nov 20;15:1722277. doi: 10.3389/fonc.2025.1722277 (PMC12676223; doi:10.3389/fonc.2025.1722277)
Supplement: Supplementary file 1 [file DataSheet1.docx]

# Supplemental material


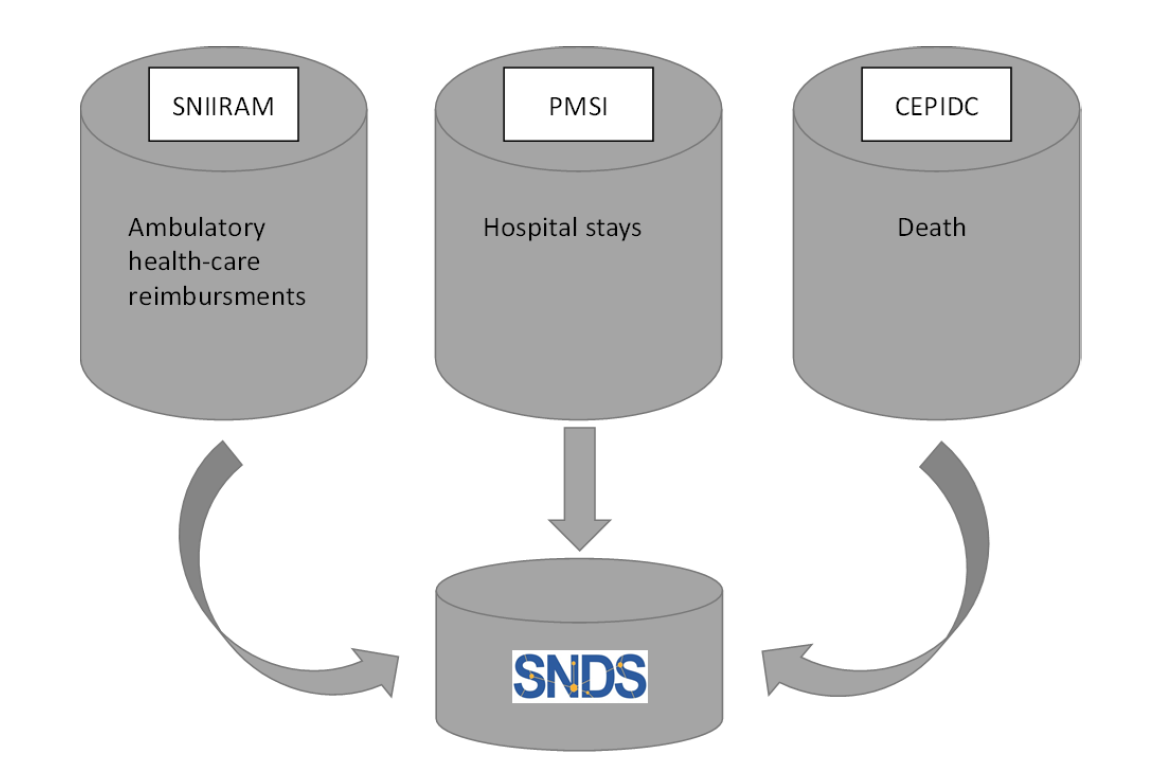


**Figure 1.** Simplified structure of SNDS. SNDS, Système National des Données de Santé (French National Health Data System); SNIIRAM, Système National d’Informations Interrégimes de l’Assurance Maladie (nationwide claims database of French public health insurance administration); PMSI, Programme de Médicalisation des Systèmes d’Information (national hospital database); CEPIDC, Centre d'Épidémiologie sur les Causes Médicales de Décès (national death registry).

Table 1. ICD-10 codes used to identify cirrhosis in the SNDS database.

| **DIAGNOSIS** | **ICD-10 CODES** |
| --- | --- |
| Alcoholic cirrhosis | K70.0, K70.2, K70.3 |
| Nonalcoholic cirrhosis | K71.7, K74.x |
| Esophageal varices with bleeding | I85.x, I98.2, I98.3 |
| Hepatic failure or encephalopathy | K70.4, K72.0, K72.1 |
| Other complications | K76.7, R18 |

Table 2. French CCAM medical procedure codes for identification of liver Doppler ultrasounds.

| **CCAM CODE** | **DESCRIPTION** |
| --- | --- |
| ZCQM001 | Transcutaneous ultrasound of the abdomen, with Doppler ultrasound of the digestive vessels |
| ZCQM002 | Transcutaneous ultrasound of the abdomen, with transcutaneous ultrasound of the pelvis and Doppler ultrasound of the digestive vessels |
| ZCQM004 | Transcutaneous ultrasound of the upper abdomen with Doppler ultrasound of the digestive vessels |
| ZCQM005 | Transcutaneous ultrasound of the abdomen, with transcutaneous ultrasound of the pelvis |
| ZCQM006 | Transcutaneous ultrasound of the upper abdomen |
| ZCQM008 | Transcutaneous ultrasound of the abdomen |
| ZCQM010 | Transcutaneous ultrasound of the upper abdomen and pelvis |
| ZCQM011 | Transcutaneous ultrasound of the upper abdomen and pelvis with Doppler ultrasound of the digestive vessels |
| HLQM001 | Transcutaneous ultrasound of the liver and bile ducts |

Table 3. ICD-10 codes used to identify alcoholic etiology of cirrhosis.

| **ICD-10 CODE** | **DESCRIPTION** |
| --- | --- |
| • E24.4 | Alcohol-induced pseudo-Cushing syndrome |
| • E51.2 | Wernicke encephalopathy |
| **E52** | **Niacin deficiency** |
| **F10** | **Mental and behavioural disorders due to use of alcohol** |
| • F10.0 | Acute intoxication |
| *F10.00* | *Without complications* |
| *F10.01* | *With trauma* |
| *F10.02* | *With other complications* |
| *F10.03* | *With delirium* |
| *F10.04* | *With perceptual distortions* |
| *F10.05* | *With coma* |
| *F10.06* | *With convulsions* |
| *F10.07* | *With pathological intoxication* |
| • F10.1 | Harmful use |
| • F10.2 | Dependence syndrome |
| *F10.20* | *Abstinent* |
| F10.200 | recent remission |
| F10.201 | partial remission |
| F10.202 | complete remission |
| *F10.21* | *Abstinent, protected environment* |
| *F10.22* | *Maintenance/substitute regime, monitored* |
| *F10.23* | *Abstinent, aversive treatment* |
| *F10.24* | *Current use* |
| F10.240 | without physical symptoms |
| F10.241 | with physical symptoms |
| *F10.25* | *Continuous use* |
| *F10.26* | *Episodic use* |
| • F10.3 | Withdrawal state |
| *F10.30* | *Without complications* |
| *F10.31* | *With convulsions* |
| • F10.4 | Withdrawal state with delirium |
| *F10.40* | *Without convulsions* |
| *F10.41* | *With convulsions* |
| • F10.5 | Psychotic disorder |
| *F10.50* | *Schizophrenic-like* |
| *F10.51* | *With delusions* |
| *F10.52* | *With hallucinations* |
| *F10.53* | *With polymorphic symptoms* |
| *F10.54* | *With depressive symptoms* |
| *F10.55* | *With manic symptoms* |
| *F10.56* | *Mixed* |
| • F10.6 | Amnesic syndrome |
| • F10.7 | Residual and late-onset psychotic disorder |
| *F10.70* | *Flashbacks* |
| *F10.71* | *Personality or behavioural disorder* |
| *F10.72* | *Residual affective disorder* |
| *F10.73* | *Dementia* |
| *F10.74* | *Other persisting cognitive impairment* |
| *F10.75* | *Late-onset psychotic disorder* |
| • F10.8 | Other mental and behavioural disorders |
| • F10.9 | Unspecified mental and behavioural disorders |
| • G31.2 | Degeneration of nervous system due to alcohol |
| • G62.1 | Alcoholic polyneuropathy |
| • I42.6 | Alcoholic cardiomyopathy |
| • K29.2 | Alcoholic gastritis |
| **K70** | **Alcoholic liver disease** |
| • K70.0 | Alcoholic fatty liver |
| • K70.1 | Alcoholic hepatitis |
| • K70.2 | Alcoholic fibrosis and sclerosis of liver |
| • K70.3 | Alcoholic cirrhosis of liver |
| • K70.4 | Alcoholic hepatic failure |
| • K70.9 | Alcoholic liver disease, unspecified |
| • K86.0 | Alcohol-induced chronic pancreatitis |
| • T51.0 | Toxic effect: ethanol |
| • Z50.2 | Alcohol rehabilitation |
| • Z71.4 | Alcohol abuse counseling and surveillance |
| • Z72.1 | Problems related to lifestyle: alcohol use |

Table 4. French CIP-13 drug codes used to identify alcoholic etiology of cirrhosis.

| **CIP-13 CODE** | **DESCRIPTION** |
| --- | --- |
| 3400930374696 | Disulfiram |
| 3400927607783 | Acamprosate |
| 3400892697789 | Baclofen |
| 3400936757219 | Naltrexone |
| 3400938861976 | Naltrexone |

Table 5. ICD-10 codes used to identify viral etiology of cirrhosis.

| **ICD-10 CODE** | **DESCRIPTION** |
| --- | --- |
| **B18** | **Chronic viral hepatitis** |
| • B18.0 | Chronic viral hepatitis B with delta agent |
| • B18.1 | Chronic viral hepatitis B without delta agent |
| • B18.2 | Chronic viral hepatitis C |
| • B18.8 | Other chronic viral hepatitis |
| • B18.9 | Chronic viral hepatitis, unspecified |

Table 6. Drug codes used to identify viral etiology of cirrhosis—hepatitis C treatments.

| **CIP-7/13**  **CODE** | **ATC CODE** | **CIP-7/13**  **CODE** | **ATC CODE** | **CIP-7/13**  **CODE** | **ATC CODE** | **CIP-7/13**  **CODE** | **ATC CODE** | **CIP-7/13**  **CODE** | **ATC CODE** |
| --- | --- | --- | --- | --- | --- | --- | --- | --- | --- |
| 3400941588211 | J05AB04 | 3400926709402 | J05AB04 | 3400935942821 | L03AB10 | 9373352 | J05AB04 | 9274408 | J05AB04 |
| 3400921604238 | J05AB04 | 3400926709341 | J05AB04 | 3400935520074 | L03AB10 | 9373398 | J05AB04 | 9298610 | J05AB04 |
| 3400941681561 | J05AB04 | 3400926709570 | J05AB04 | 3400935518934 | L03AB10 | 9373346 | J05AB04 | 9373398 | J05AB04 |
| 3400941552779 | J05AB04 | 3400935197191 | J05AB04 | 3400935519184 | L03AB10 | 9373406 | J05AB04 | 9367587 | J05AB04 |
| 3400941552311 | J05AB04 | 3400941684524 | J05AB04 | 3400935943712 | L03AB10 | 9208570 | J05AB04 | 9373381 | J05AB04 |
| 3400941551949 | J05AB04 | 3400936586680 | J05AB04 | 3400935943361 | L03AB10 | 9250187 | J05AB04 | 9351451 | J05AB04 |
| 3400941551598 | J05AB04 | 3400936200395 | J05AB04 | 3400935942999 | L03AB10 | 9298610 | J05AB04 | 9369126 | J05AB04 |
| 3400937484725 | J05AB04 | 3400935197252 | J05AB04 | 3400935942470 | L03AB10 | 36777 | J05AB04 | 9408949 | J05AB04 |
| 3400941588150 | J05AB04 | 3400926625382 | J05AB04 | 3400935941930 | L03AB10 | 9373381 | J05AB04 | 9362940 | J05AE11 |
| 3400939990569 | J05AB04 | 3400926625153 | J05AB04 | 3400935942012 | L03AB10 | 9274408 | J05AB04 | 9397772 | J05AE14 |
| 3400941683114 | J05AB04 | 3400922268149 | J05AB04 | 3400935943422 | L03AB10 | 9362940 | J05AE11 | 9401108 | J05AE14 |
| 3400941684753 | J05AB04 | 3400921695885 | J05AB04 | 3400935519474 | L03AB10 | 9373122 | J05AE12 | 9399742 | J05AX14 |
| 3400941682742 | J05AB04 | 3400921695656 | J05AB04 | 3400935520883 | L03AB10 | 9222854 | L03AB09 | 9399759 | J05AX14 |
| 3400941682391 | J05AB04 | 3400937484664 | J05AB04 | 3400935521194 | L03AB10 | 9222021 | L03AB10 | 9402846 | J05AX14 |
| 3400941682162 | J05AB04 | 3400936159143 | J05AB04 | 3400935521316 | L03AB10 | 9241892 | L03AB10 | 9402852 | J05AX14 |
| 3400937327787 | J05AB04 | 3400921695717 | J05AB04 | 3400935520654 | L03AB10 | 9241917 | L03AB10 | 9398211 | J05AX15 |
| 3400936159082 | J05AB04 | 3400921604177 | J05AB04 | 3400921716801 | L03AB11 | 9221984 | L03AB10 | 9221984 | L03AB10 |
| 3400936200456 | J05AB04 | 3400927327537 | J05AB04 | 3400921717341 | L03AB11 | 9241863 | L03AB10 | 9222015 | L03AB10 |
| 3400935197313 | J05AB04 | 3400926709280 | J05AB04 | 3400935995988 | L03AB11 | 9222015 | L03AB10 | 9222021 | L03AB10 |
| 3400941681912 | J05AB04 | 3400927901010 | J05AB04 | 3400935996121 | L03AB11 | 9241886 | L03AB10 | 9241047 | L03AB11 |
| 3400927901249 | J05AB04 | 3400921924985 | J05AE11 | 3400935995810 | L03AB11 | 9241900 | L03AB10 | 9241076 | L03AB11 |
| 3400937779531 | J05AB04 | 3400921737851 | J05AE11 | 3400935996060 | L03AB11 | 9221990 | L03AB10 | 9241863 | L03AB10 |
| 3400926625214 | J05AB04 | 3400941946790 | J05AE12 | 3400926980511 | L03AB11 | 9222009 | L03AB10 | 9241886 | L03AB10 |
| 3400939990101 | J05AB04 | 3400935214492 | L03AB09 | 3400921716979 | L03AB11 | 9374682 | L03AB11 | 9241892 | L03AB10 |
| 3400937779760 | J05AB04 | 3400935214553 | L03AB09 | 3400921718171 | L03AB11 | 9374699 | L03AB11 | 9241900 | L03AB10 |
| 3400941682803 | J05AB04 | 3400935214614 | L03AB09 | 9367587 | J05AB04 | 9241047 | L03AB11 | 9241917 | L03AB10 |
| 3400941588389 | J05AB04 | 3400935519764 | L03AB10 | 9373412 | J05AB04 | 9241076 | L03AB11 | 9374682 | L03AB11 |
| 3400941684463 | J05AB04 | 3400935943880 | L03AB10 | 9369126 | J05AB04 | 9395388 | L03AB11 | 9374699 | L03AB11 |
| 3400941588440 | J05AB04 | 3400935942302 | L03AB10 | 9351451 | J05AB04 | 9208570 | J05AB04 | 9395388 | L03AB11 |
| 3400939990279 | J05AB04 | 3400935520364 | L03AB10 | 9355101 | J05AB04 | 9250187 | J05AB04 |  |  |

Table 7. Drug codes used to identify viral etiology of cirrhosis—hepatitis B treatments.

| **CIP-7/13 CODE** | **ATC CODE** |
| --- | --- |
| 3400936156760 | J05AF08 |
| 3400937629119 | J05AF10 |
| 3400937628976 | J05AF10 |
| 3400937629287 | J05AF10 |
| 3400937993579 | J05AF11 |
| 9246642 | J05AF08 |
| 9288327 | J05AF10 |
| 9288310 | J05AF10 |
| 9288304 | J05AF10 |
| 9300956 | J05AF11 |
| 9246642 | J05AF08 |
| 9288304 | J05AF10 |
| 9288310 | J05AF10 |
| 9288327 | J05AF10 |
| 9212525 | J05AF05 |
| 9212531 | J05AF05 |
| 3400935196712 | J05AF05 |
| 3519671 | J05AF05 |
| 3400935196941 | J05AF05 |
| 3519694 | J05AF05 |
| 3400926789183 | J05AF07 |
| 3400935850010 | J05AF07 |
| 3400926789015 | J05AF07 |
| 3400926788933 | J05AF07 |
| 3400926788872 | J05AF07 |
| 9393573 | J05AF07 |
| 9236247 | J05AF07 |
| 9393567 | J05AF07 |
| 9393550 | J05AF07 |
| 9393544 | J05AF07 |
| 9236247 | J05AF07 |
| 9393573 | J05AF07 |
| 9393544 | J05AF07 |
| 9393550 | J05AF07 |
| 9393567 | J05AF07 |
| 9183195 | J05AF05 |
| 9183203 | J05AF05 |
| 9239228 | J05AF05 |
| 9386573 | J05AF05 |
| 9386596 | J05AF05 |

Table 8. Codes used in Charlson Index construction

|  | ICD-10 codes (in-hospital^1^ or LTD diagnoses) | | | Medical procedures^1^ | | | Medication^1^ |  |
| --- | --- | --- | --- | --- | --- | --- | --- | --- |
| Myocardial infarction | I21;I22;I252;I255 |  |  | |  |  |  |  |
| Congestive heart failure | I110;I130;I132;I50 | | |  | |  | |  |
| Peripheral vascular disease | I70;I71;I731;I738;I739;I771;I790;I792;K551; K558;K559; Z958;Z959 | | | peripheral vascular stenting | |  | |  |
| Cerebrovascular disease | G45;G46;H340;I60-I69 | | |  | |  | | |
| Dementia | F00-F03;F051;G30;G311 | | |  | | at least 3 reimbursements of anti-Alzheimer drugs | | |
| Chronic pulmonary disease | I278;I279;J40-J47;J60-J67;J684;J701;J703 | | |  | | at least 2 reimbursements of bronchodilator drugs | | |
| Connective tissue disease | M05;M06;M315;M32;M33;M34;M351;M353;M360 | | |  | |  | | |
| Ulcer disease | K25-K28 | | |  | |  | | |
| Mild liver disease | B18;K700-K703;K709;K713K715;K717;K73; K74;K760; K762-K764;K768;K769;Z944 | | |  | |  | | |
| Diabetes | E100;E101;E106;E108;E109;E110;E111;E116; E118;E119; E120;E121; E126;E128-E131;E136;  E138-E141; E146;E148; E149 | | |  | | at least 3 reimbursements (or at least 2 in case of large pack sizes) of oral antidiabetic agents and/or insulin | | |
| Hemiplegia | G041;G114;G801;G802;G81;G82; G830;  G831-G834;G839 | | |  | |  | |  |
| Moderate or severe renal disease | I120;I131;N032-N037;N052-N057;N18;N19; N250;Z490;Z491; Z492;Z940;Z992 | | | dialysis | |  | |  |
| Diabetes with end-organ damage^2^ | E102-E105;E107;E112-E115;E117; E122-E125; E127;E132-E135;E137; E142-E145;E147 | | | laser surgery for diabetic retinopathy | |  | |  |
| Any tumor (including lymphoma and leukemia except for malignant neoplasm of skin) | C00-C26;C30-C34;C37-C41;C43; C45-C58;  C60-C76;C81-C85;C88; C90-C97 | | |  | |  | |  |
| Moderate or severe liver disease | I850;I859;I864;I982;K704;K711; K721;K729;  K765-K767 | | |  | |  | |  |
| Metastatic solid tumor | C77-C80 | | |  | |  | |  |
| HIV-AIDS | B20-B22;B24;Z21 | | |  | |  | |  |

| Table 9. Charlson Index - Codes extracted from the CCAM corresponding to vascular stent graft or heart valve procedures | | | | | | |
| --- | --- | --- | --- | --- | --- | --- |
| DBKA001 | EAAF002 | EEAF006 |  |  |  |  |
| DBKA002 | EAAF900 | EELF002 |  |  |  |  |
| DBKA003 | EAAF902 | EEPF001 |  |  |  |  |
| DBKA004 | EBAF001 | EFAF001 |  |  |  |  |
| DBKA005 | EBAF006 | EFLF001 |  |  |  |  |
| DBKA006 | EBAF010 | EFPF001 |  |  |  |  |
| DBKA007 | EBAF011 | EGAF002 |  |  |  |  |
| DBKA008 | EBAF014 | EGAF004 |  |  |  |  |
| DBKA009 | ECAF001 | EGPF001 |  |  |  |  |
| DBKA010 | ECAF004 | EHAF001 |  |  |  |  |
| DBKA011 | ECLF003 | EHAF004 |  |  |  |  |
| DBKA012 | ECLF004 | EHCF002 |  |  |  |  |
| DDAF003 | ECPF001 | ENAF001 |  |  |  |  |
| DDAF004 | ECPF002 | EPPF003 |  |  |  |  |
| DDAF006 | ECPF005 | EZAF002 |  |  |  |  |
| DDAF007 | EDAF001 | EZJF001 |  |  |  |  |
| DDAF008 | EDAF003 | EZPF003 |  |  |  |  |
| DDAF009 | EDAF005 |  |  |  |  |  |
| DDPF002 | EDAF006 |  |  |  |  |  |
| DFAF001 | EDAF010 |  |  |  |  |  |
| DFAF003 | EDLF004 |  |  |  |  |  |
| DGAF003 | EDLF005 |  |  |  |  |  |
| DGAF004 | EDLF006 |  |  |  |  |  |
| DGAF005 | EDLF007 |  |  |  |  |  |
| DGAF007 | EDLF008 |  |  |  |  |  |
| DGLF001 | EDLF013 |  |  |  |  |  |
| DGLF002 | EDPF001 |  |  |  |  |  |
| DGLF005 | EDPF004 |  |  |  |  |  |
| DGLF012 | EDPF005 |  |  |  |  |  |
| DGPF002 | EDPF006 |  |  |  |  |  |
| DHAF001 | EDPF009 |  |  |  |  |  |
| DHAF004 | EEAF002 |  |  |  |  |  |
| DHPF002 | EEAF004 |  |  |  |  |  |

| Table 10. Charlson Index - Codes extracted from the CCAM corresponding to an extrarenal purification session | | | | | |
| --- | --- | --- | --- | --- | --- |
| JVJB001 | JVJF007 |  |  |  |  |
| JVJB002 | JVJF008 |  |  |  |  |
| JVJF002 | JVRP004 |  |  |  |  |
| JVJF003 | JVRP007 |  |  |  |  |
| JVJF004 | JVRP008 |  |  |  |  |
| JVJF005 | YYYY007 |  |  |  |  |

| Table 11. Charlson Index - CCAM codes corresponding to laser treatment for diabetic retinopathy | | | | | |
| --- | --- | --- | --- | --- | --- |
| BGMA002 |  |  |  |  |  |
| BGNP003 |  |  |  |  |  |

Data cleaning - Code used

options compress=yes;

/********** ALD06 **********/

**proc** **sql**;

%***connectora***;

create table SAM.patients_ALD06 as select * from connection to oracle

(select distinct a.ben_nir_psa

from ir_cim_v b,

ir_imb_r a

where trim(a.med_mtf_cod) = trim(b.cim_cod)

and a.imb_ald_dtd <= to_date('31/12/2015','dd/mm/yyyy')

and (a.imb_ald_dtf >= to_date('01/01/2007','dd/mm/yyyy') or a.imb_ald_dtf = to_date('01/01/0001','dd/mm/yyyy'))

and b.ald_030_cod in('06')

);

disconnect from oracle;

**quit**;

**%macro** CIRR(Ann=);

%if &Ann. < **08** %then %do;

proc sql;

%***connectora***;

create table SAM.CIM_CIRR_&Ann. as select * from connection to oracle

(select distinct c.nir_ano_17 as ben_nir_psa, b.dgn_pal, d.ass_dgn

from

t_mco&Ann.c c,

t_mco&Ann.b b,

t_mco&Ann.d d

where b.eta_num = c.eta_num

and b.rsa_num = c.rsa_num

and c.eta_num = d.eta_num (+)

and c.rsa_num = d.rsa_num (+)

/* Elimination of false ID (NIR)- 1*/

and c.nir_ret = '0' and c.nai_ret = '0' and c.sex_ret = '0' and c.sej_ret = '0'

and c.fho_ret = '0' and c.pms_ret = '0' and c.dat_ret = '0'

/* Elimination of fictional stays (Etbs EX-OQN)*/

and (b.grg_ghm not in ('90Z00Z') and b.grg_ret not in ('24'))

/* Elimination of error stay reports (GHM)*/

and b.grg_ghm not in ('90H01Z','90Z00Z','90Z01Z','90Z02Z','90Z03Z')

/* Elimination of center duplicated IDs (APHP, APHM, HCL */

and b.eta_num not in (

'130780521', '130783236', '130783293', '130784234', '130804297', '600100101', '750041543',

'750100018', '750100042', '750100075', '750100083', '750100091', '750100109', '750100125',

'750100166', '750100208', '750100216', '750100232', '750100273', '750100299', '750801441',

'750803447', '750803454', '910100015', '910100023', '920100013', '920100021', '920100039',

'920100047', '920100054', '920100062', '930100011', '930100037', '930100045', '940100027',

'940100035', '940100043', '940100050', '940100068', '950100016', '690783154', '690784137',

'690784152', '690784178', '690787478', '830100558')
